# Outside-In Signaling as a Detonator-like Stimulus of Integrin Activation by Promoting Both Affinity and Avidity

**DOI:** 10.3390/biom16091358

**Published:** 2026-09-18

**Authors:** Naoyuki Kondo, Tatsuo Kinashi

**Affiliations:** Department of Molecular Genetics, Institute of Biomedical Science, Kansai Medical University, Hirakata 573-1010, Osaka, Japan; kinashi.tat@kmu.ac.jp

**Keywords:** integrin, outside-in signaling, LFA1, affinity, avidity, LC3, LAPTIN, talin1, kindlin-3

## Abstract

Integrins play a central role in a wide range of biological processes, including development, tissue homeostasis, immune responses, and disease progression. Their activation is traditionally explained by two major signaling modes: inside-out signaling, which converts integrins to ligand-competent and high-affinity states through intracellular activators, including the Rap1-talin-kindlin axis, and outside-in signaling, which transduces ligand engagement into cytoskeletal coupling and downstream signaling. Recent findings indicate that this division is incomplete. Ligand-engaged integrins can also generate spatially organized signaling platforms that feed back to enhance integrin adhesiveness *per se*. In this revised view, outside-in signaling does not merely report the completion of adhesion but can amplify both affinity, by stabilizing activator binding and high-affinity conformations, and avidity, by promoting integrin clustering and local integrin accumulation. This feedback mechanism is most extensively characterized in lymphocytes, where LFA1 (αLβ2) outside-in signaling activates Rap1 and recruits talin1 and kindlin-3, increases LFA1-ICAM1 binding stability, and promotes LFA1 transport through ATG8/LC3-associated machinery. Importantly, this ATG8/LC3-dependent process, named LAPTIN, does not represent canonical degradative autophagy but rather defines an integrin-induced LC3-associated protein transport pathway that promotes LFA1 accumulation. These observations position outside-in signaling as a potential “detonator” of positive-feedback activation rather than a passive consequence of ligand binding. In this review, we summarize the structural and cellular basis of affinity and avidity regulation, compare activation-promoting outside-in and related feedback mechanisms across adherent-cell, leukocyte, and platelet systems, and discuss how outside-in–based trafficking, LC3-associated protein transport, metabolic signaling, and mechanical force reveal shared regulatory principles alongside integrin-specific implementations, with potential implications for therapeutic targeting.

## 1. Introduction

Integrins are membrane receptors composed of α and β subunits that couple cells to the extracellular matrix (ECM) and to neighboring cells. These interactions are essential for organizing multicellular homeostasis, such as morphogenesis [1,2], epithelial and muscular integrity [3,4,5,6,7,8], hemostasis [9,10], and immune cell positioning [11,12,13]. These functions depend not only on adhesion but also on the ability of ligand-engaged integrins to initiate outside-in and mechanochemical signaling that reorganizes the actin cytoskeleton, orients cell polarity, couples ECM cues to transcription, promotes survival, and, in immune cells, supports effector responses [5,10,14,15,16,17]. Thus, to understand integrin physiology more deeply, it is not sufficient to determine which ligands integrins bind or where these ligands are expressed. It is also necessary to ask how ligand binding is converted into intracellular signals and, conversely, how these signals feed back to regulate integrin adhesiveness itself. In this review, we first summarize the conventional framework of integrin regulation, then discuss emerging evidence that outside-in signaling can potentiate integrin activation, and finally consider the therapeutic implications of this revised view.

### 1.1. Affinity and Avidity

**Figure 1:**
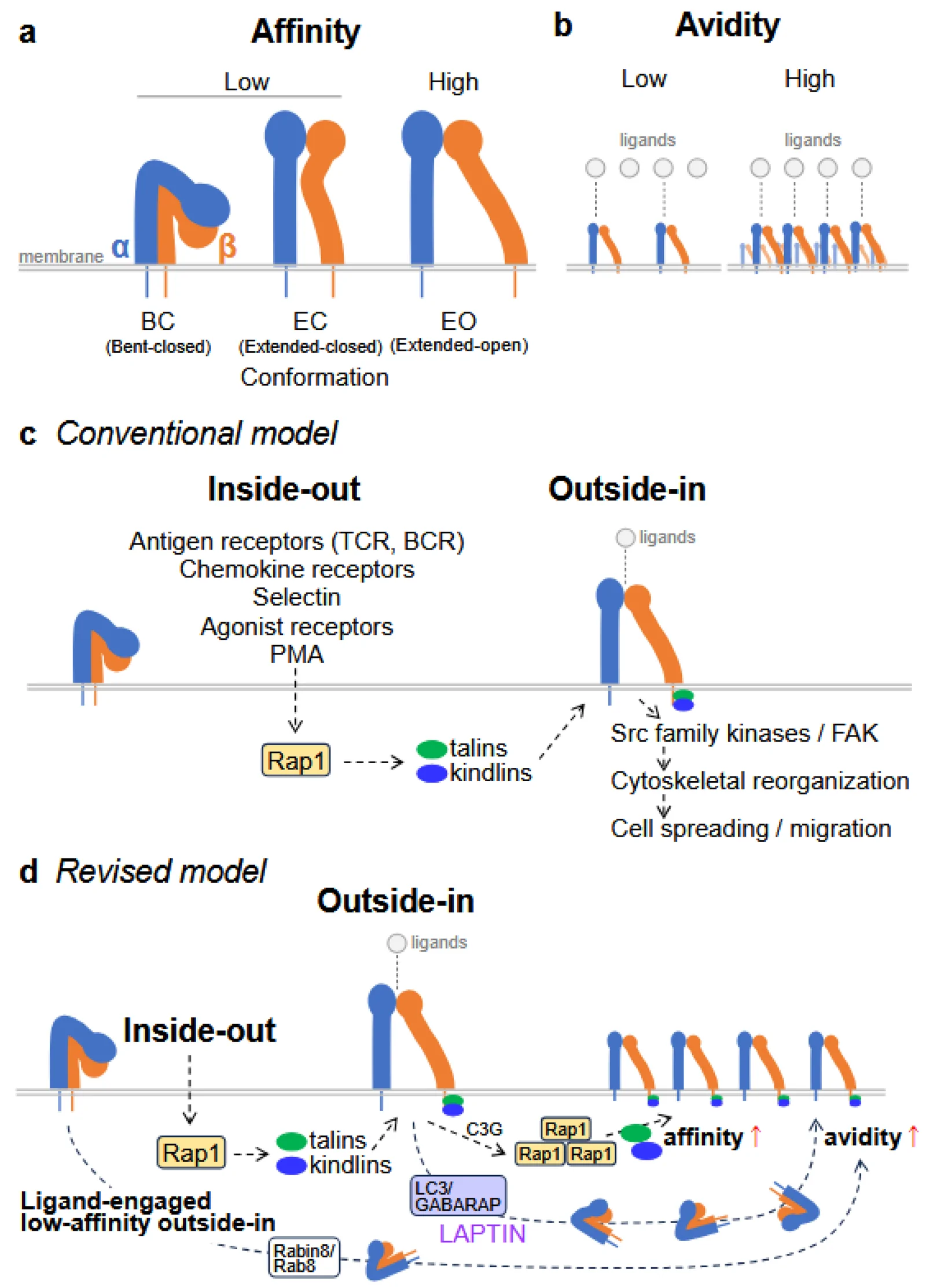
Conventional and revised concepts of integrin activation. (**a**) Integrin affinity is controlled by conformational equilibria among the bent-closed (BC), extended-closed (EC), and extended-open (EO) states. Transition from the BC or EC state to the EO state involves headpiece opening and confers high ligand-binding affinity. (**b**) Integrin avidity reflects the effective adhesive strength generated at the cell surface and is increased by receptor accumulation, lateral organization, and multivalent ligand engagement. (**c**) A conventional model of integrin activation. Inside-out signaling downstream of antigen receptors [T cell receptor (TCR) and B cell receptor (BCR)], chemokine receptors, selectin-associated signals, agonist receptors, or PMA activates the Rap1-talin-kindlin axis to promote ligand-competent integrins. Conversely, ligand engagement initiates outside-in signaling through Src family kinases, focal adhesion kinase (FAK), and related effectors, leading to cytoskeletal reorganization, cell spreading, and migration. (**d**) A revised model of integrin activation. In the revised model emphasized in this review, ligand-engaged high-affinity LFA1 promotes C3G membrane recruitment and Rap1 activation, reinforcing talin/kindlin engagement and integrin affinity, while also engaging the ATG8/LC3/GABARAP-associated **L**C3 **A**ssociated **P**rotein **T**ransport induced by **I**ntegri**N** (LAPTIN) machinery to reinforce avidity. Ligand-engaged low-affinity LFA1-dependent outside-in signaling also promotes Rabin8-Rab8 axis-dependent LFA1 accumulation and avidity. Dashed arrows indicate signaling or trafficking relationships. Upward red arrows indicate increases in affinity or avidity.

Integrin adhesiveness is controlled by two separable but cooperative parameters: affinity and avidity. Affinity refers to the intrinsic ligand-binding strength of an individual integrin heterodimer. Structural and biophysical studies have indicated that integrins exist in three major conformational states: bent-closed (BC), extended-closed (EC), and extended-open (EO) [18,19,20,21] (Figure 1a). Importantly, both the BC and EC states retain a closed-headpiece structure and intrinsically low affinity, whereas the headpiece opening in the EO state markedly increases the ligand affinity [20,21]. In α5β1, direct measurements of intrinsic affinities showed that the EO state has more than 5000-fold higher affinity for ligands than the BC and EC states, indicating that headpiece opening, rather than extension alone, is the decisive structural step for high-affinity ligand binding [21]. Biophysical analysis further indicates that affinity regulation reflects both conformational-state populations and the intrinsic affinity within each state; for α5β1, metal occupancy at the MIDAS/ADMIDAS modulates both parameters [22]. In leukocytes, the functional importance of this transition is underscored by the finding that the intermediate-affinity LFA1 (αLβ2) state supports neutrophil slow rolling, whereas the high-affinity open-headpiece conformation is required for firm arrest [23]. Single-molecule analysis of LFA1-ICAM1 interactions further showed that the stabilization of high-affinity LFA1 prolongs ligand-binding events at the single-molecule level in live lymphocytes [24]. More recently, single-molecule FRET analysis of full-length α5β1 integrin showed that ligand binding to the predominant BC state can itself initiate a concerted transition to leg extension and headpiece opening within milliseconds, whereas unliganded conformational transitions occur much more slowly [25]. This ligand-first pathway is best viewed as a mechanistic refinement of, rather than a replacement for, the long-standing concept of bidirectional integrin signaling. Ligand occupancy was already shown to induce a high-affinity fibrinogen-binding state of αIIbβ3 and platelet aggregation in early work [26]. The single-molecule FRET study advances this concept by resolving the order and kinetics of individual conformational transitions: ligand binding to BC α5β1 precedes a rapid concerted transition in which leg extension and headpiece opening occur together, with EC not serving as an obligatory intermediate [25]. Thus, the advance lies primarily in the temporal and structural resolution of ligand-induced allostery rather than in the general concept that ligands can activate integrins. To date, this ligand-first kinetic sequence has not yet been directly demonstrated by single-molecule FRET for LFA1. In lymphocyte and lymphoid cell systems, complementary approaches have shown ligand-dependent conformational changes: immobilized ICAM1 promoted an open-headpiece LFA1 conformation in TCR-stimulated primary human lymphocytes [27], iPALM demonstrated LFA1 extension in ICAM1-engaged Jurkat T cells [28], and an extremely high concentration of ICAM1 induced exposure of the EO-specific mAb24 epitope in LFA1-expressing lymphocytes [29]. Whether this rapid ligand-first BC-to-EO pathway, also observed for α5β1, operates similarly in LFA1 during physiological lymphocyte activation therefore remains an open question. Functionally, this conformational plasticity allows integrins to act as switch-like adhesion receptors, particularly in leukocytes, which must remain weakly adhesive at steady state but respond rapidly upon stimulation and ligand binding.

Avidity refers to the effective adhesive strength generated at the cell surface, and is influenced not only by receptor number but also by lateral mobility, local receptor enrichment, and multivalent rebinding [30,31] (Figure 1b). Recent mechanistic studies have begun to define this layer more directly. Active talin can promote integrin microclustering through talin dimerization, with paxillin and kindlin strengthening the complex and enhancing binding to multivalent ligands [32]. In a reconstituted αIIbβ3 system, talin and kindlin independently induced clustering and acted synergistically to form larger clusters, with kindlin increasing integrin and talin density [33]. In leukocytes and other rapidly responding cells, these mechanisms help concentrate productive ligand-bound integrins at sites of contact, thereby stabilizing adhesion under dynamic conditions, including shear flow [34,35] and immunological synapse formation [23]. However, compared with affinity regulation, the molecular framework governing avidity regulation is less unified and appears to be more context-dependent [30,31]. This relative gap raises an important question: how are ligand-engaged integrins actively reorganized at the cell surface to enhance their avidity? This topic is revisited in the context of a new aspect of outside-in signaling. As discussed below, outside-in signaling provides a route by which ligand binding can reorganize integrins, bridging the conceptual gap between conformational activation and spatial amplification.

### 1.2. Inside-Out and Outside-In Signaling

Integrin activation is controlled by bidirectional signaling pathways, namely, the inside-out and outside-in signaling pathways (Figure 1c). Inside-out signaling is initiated by diverse upstream receptors, including chemokine and antigen receptors in leukocytes, selectin-associated signaling during leukocyte rolling, and agonist receptors for thrombin, ADP, and thromboxane A2 in platelets; phorbol esters, such as phorbol 12-myristate 13-acetate (PMA), can experimentally mimic some of these pathways (Figure 1c, left) [36,37,38]. These inputs converge on the Rap1-talin-kindlin activation axis [36,37]. In leukocytes, the LFA1-relevant core components of this axis are Rap1, talin1, and kindlin-3 [23,39]. Activated Rap1 promotes talin1 recruitment to the plasma membrane directly and/or, particularly in hematopoietic cells, through the Rap1 effector RIAM, thereby facilitating talin1 binding to the integrin β cytoplasmic tail (CT) [39,40,41,42,43]. However, under mAb24-stimulated conditions, in which the LFA1 EO state is directly stabilized, we showed that RIAM deficiency did not measurably reduce talin1-binding kinetics to the β2-CT, suggesting that RIAM is not the sole determinant of talin1 engagement once EO-dependent positive feedback has been established [39]. Talin binding destabilizes the α/β CT clasp and favors integrin extension, whereas kindlins cooperate with talin1 to promote and/or stabilize the high-affinity open conformation [43,44]. Recent work further showed that talin and kindlin binding can allosterically remodel the β-integrin cytoplasmic tail, increasing talin affinity while decreasing kindlin affinity and enabling a dynamic ternary talin–β-integrin–kindlin complex [45]. For LFA1, single-molecule analysis recently showed that kindlin-3 can disrupt an α/β CT clasp and promote EO formation [39]. The net effect of inside-out signaling is to shift integrins from low-adhesive conformations to ligand-competent states, priming cells for rapid adhesion under physiological conditions.

In contrast, outside-in signaling is initiated after ligand engagement and converts physical binding cues into intracellular responses by promoting cytoskeletal coupling, integrin reorganization at the site of contact, and recruitment of signaling molecules, such as Src family kinases, FAK, and other adhesion-associated effectors (Figure 1c, right) [14,46]. Through FAK/Src-dependent phosphorylation of adhesion-associated adaptors, such as paxillin and p130Cas, and through crosstalk with Rho-family GTPases, ligand-engaged integrins regulate Rac1/Cdc42-dependent protrusive actin assembly, RhoA-dependent actomyosin contractility, focal adhesion maturation, and cell migration [14,46,47,48,49]. This conventional view places outside-in signaling downstream of ligand binding and adhesion strengthening. The studies discussed below extend this view by suggesting that outside-in signaling feeds back to promote integrin activation itself, thereby reinforcing both affinity and avidity at sites of ligand engagement (Figure 1d).

## 2. Outside-In Signaling Links to Integrin Activation Machinery

Outside-in signaling is increasingly recognized to influence additional layers of integrin regulation. In particular, accumulating evidence suggests that outside-in signaling can potentiate integrin activation itself. In LFA1, high-affinity conformation-dependent outside-in signaling promotes the accumulation and binding of canonical inside-out activators, including Rap1, talin1, and kindlin-3, and enhances the recruitment of LFA1 to the contact sites [39]. In this section, we summarize historically dispersed observations that point to an activation-promoting role of outside-in signaling and discuss their potential conceptual importance.

### 2.1. Focal Adhesion Complexes in Adherent Cells

**Figure 2:**
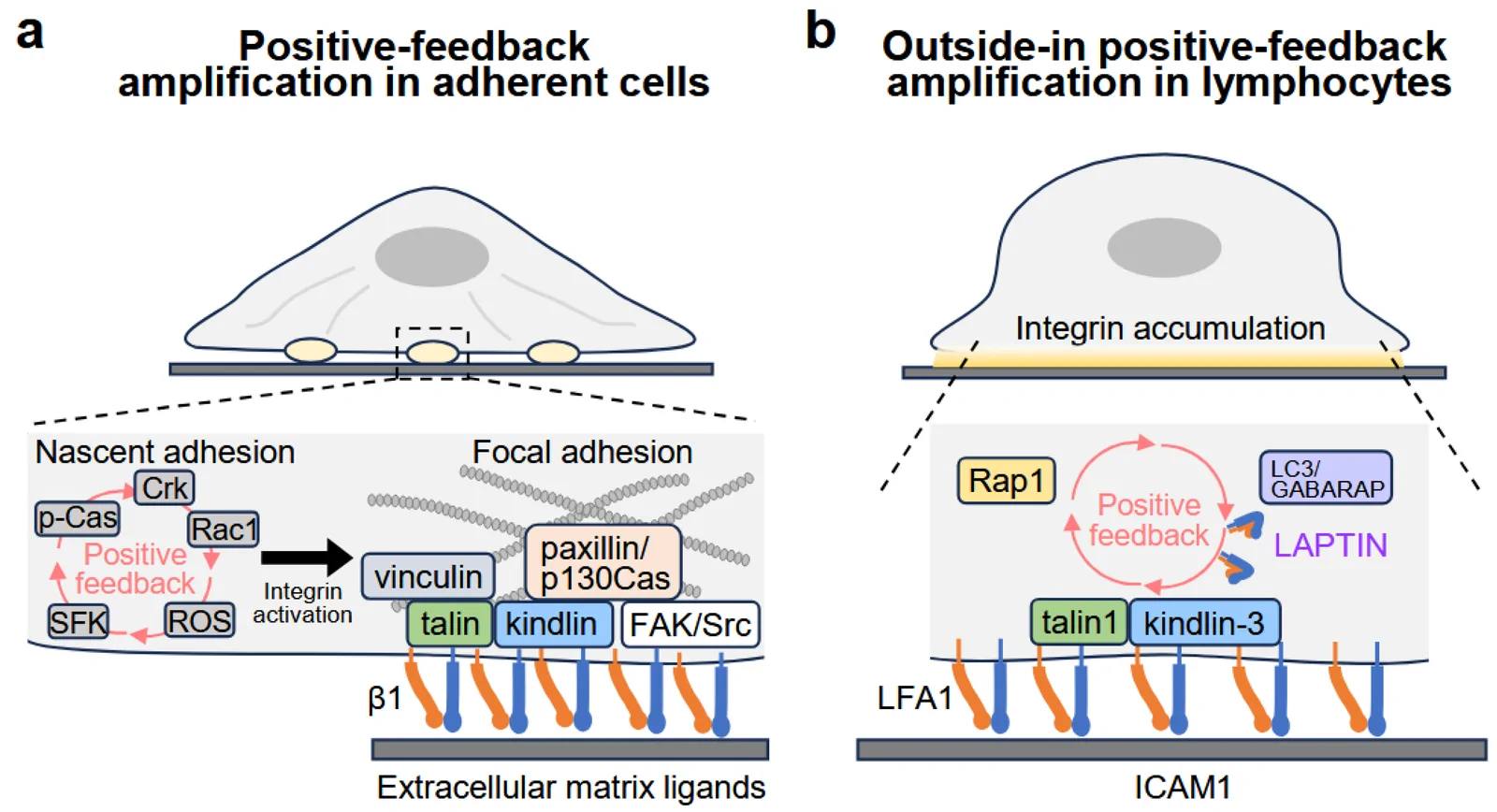
Positive-feedback amplification in adherent cells and lymphocytes. (**a**) In adherent cells, an SFK–p-Cas–Crk–Rac1–ROS positive-feedback loop operates during nascent adhesion assembly and precedes integrin activation and focal adhesion maturation. Mature focal adhesions contain talin, kindlin, vinculin, paxillin/p130Cas, FAK, and Src family kinases and function as local signaling and mechanotransduction platforms. (**b**) In lymphocytes, ligand-engaged lymphocyte function-associated antigen 1 (LFA1) forms transient contact-site activation hubs rather than large, long-lived focal adhesions. LFA1 outside-in signaling promotes Rap1 activation and talin1/kindlin-3 recruitment and supports integrin accumulation through the ATG8/LC3/GABARAP-associated LAPTIN machinery. Together, these processes provide mechanisms by which ligand engagement amplifies both integrin affinity and integrin avidity at the immune-cell contact interface. Intercellular adhesion molecule 1 (ICAM1) is shown as a representative LFA1 ligand.

In adherent cells, focal adhesions represent canonical sites of integrin outside-in signaling, where ligand-engaged integrins assemble multiprotein adhesion complexes and transmit biochemical and mechanical signals to the actin cytoskeleton [50,51]. In this view, focal adhesions are not merely structural anchors but also signaling platforms that couple ECM binding to cytoskeletal organization, adhesion maturation, mechanotransduction, and downstream responses [46,52,53,54]. Within these clusters, integrins are enriched in active conformations. Conformation-sensitive imaging has shown that extended α5β1 integrins are enriched in focal adhesions, whereas super-resolution analyses have revealed that active and inactive β1 integrins are localized in distinct nanoclusters within focal adhesions [55,56]. In parallel, talin1-vinculin interactions have been shown to promote the clustering of activated integrins and increase their residency time in focal adhesions, whereas kindlin can stabilize the talin-integrin bond under mechanical load, supporting the idea that focal adhesions can function as local platforms that stabilize active integrin states (Figure 2a) [57,58]. Single-particle tracking and quantitative single-molecule imaging further indicate that kindlin membrane diffusion/localization and talin-kindlin-integrin complex formation are tightly linked to integrin activation and focal adhesion assembly [59,60]. This body of evidence indicates that focal adhesions can spatially concentrate active integrins and integrin adaptors, thereby stabilizing the activated integrin state (Figure 2a). Recent work has added a temporal dimension to this model. Phosphorylated Cas clusters containing inactive β1 integrin can form before β1 activation, and an SFK-Cas-Crk-Rac1-ROS positive-feedback loop promotes growth of these nascent adhesions before recruitment of talin, kindlin, vinculin, and other core focal adhesion components (Figure 2a) [61]. The temporal ordering is consistent with feedback amplification during early adhesion assembly before focal adhesion maturation; however, it has not yet been fully established whether high-affinity integrin formation itself acts as a driving force for the accumulation of integrin adaptors and integrins. This unsolved point has become particularly important in light of recent findings on leukocyte integrins, as discussed below in this section.

### 2.2. Integrin Activation and Amplification in Leukocytes and Platelets

Leukocytes and platelets provide a complementary setting to classical focal adhesions because, during physiological adhesion, they typically rely on rapid and dynamically organized integrin-mediated contacts rather than the large, long-lived focal adhesions characteristic of many adherent cells [62,63,64]. Resting circulating leukocytes and platelets maintain low basal adhesiveness to avoid inappropriate adhesion, but must rapidly establish productive contacts after chemokine or antigen-receptor stimulation, vascular injury, or other activating cues. Leukocytes form structures such as immunological synapses and integrin-rich adhesion zones, whereas platelets rapidly form adhesive contacts and aggregates during hemostasis [63,65,66,67]. This operational feature makes lymphocytes and platelets useful for testing whether outside-in signaling merely follows adhesion to induce cytoskeletal reorganization or can instead feed back directly into the integrin activation machinery. The previous single-molecule analysis of the LFA1-ICAM1 interaction, the first in situ measurement of integrin binding time in live cells, provides an important starting point for this question: stabilization of high-affinity LFA1 prolongs the lifetime of LFA1-ICAM1 binding events [24]. However, because avidity depends on the number and spatial organization of productive binding events at the contact plane [30,31,34], the relationship between high-affinity LFA1 formation and avidity regulation remains to be elucidated. More generally, if ligand engagement of high-affinity integrin also increases the local integrin density, outside-in signaling would represent an amplifier that links affinity and avidity.

Studies of LFA1 support this hypothesis. Formation of the LFA1 EO conformation triggers outside-in signaling that activates Rap1 and causes marked accumulation of talin1 and kindlin-3 at the cell contact plane [39]. Importantly, this process is not simply a downstream consequence of pre-existing inside-out activation. EO-dependent outside-in signaling increases both the frequency and lifetime of talin1 and kindlin-3 interactions with the LFA1 β2-CT, thereby favoring further LFA1 activation and stabilization of the high-affinity state [39]. This observation addresses, in a lymphocyte context, the question raised above for focal adhesions: ligand-engaged high-affinity integrins can positively feed back to the same Rap1-talin1-kindlin-3 module that is usually regarded as the executor of inside-out signaling (Figure 1d and Figure 2b).

In parallel, outside-in signaling also contributes to LFA1 avidity. LFA1 outside-in signaling promotes LFA1 accumulation at the contact plane [39,68]. Concomitant with LFA1 accumulation, LFA1 forms intracellular clusters. Notably, low-affinity LFA1 can induce a moderate increase in LFA1 accumulation, whereas high-affinity EO LFA1 more potently promotes LFA1 intracellular clustering and accumulation at the cell contact plane [68,69]. These findings suggest that lymphocytes can generate transient, spatially organized activation hubs that are distinct from classical focal adhesions but conceptually similar in their ability to concentrate active integrins, trafficking regulators, and integrin-associated signaling modules (Figure 2b).

Collectively, in LFA1, these observations suggest that outside-in signaling enhances integrin adhesiveness at two levels. First, outside-in signaling reinforces affinity by recruiting and stabilizing the interactions between integrins and the canonical intracellular activators. Second, it reinforces avidity by promoting LFA1 clustering and contact-site accumulation. In this sense, outside-in signaling behaves as an amplifier of the inside-out signaling machinery and the spatial organization of integrins. On this basis, ligand engagement can serve as a “detonator” that initiates positive feedback loops for integrin activation, rather than merely reporting the endpoint of inside-out signaling (Figure 1d and Figure 2b), as previously believed.

A conceptually related amplification is observed in platelets. Early work showed that occupancy of αIIbβ3 by short RGD ligands can induce a high-affinity fibrinogen-binding state, demonstrating ligand-induced allosteric activation, although this observation alone does not establish a cytoplasmic outside-in feedback loop [26]. More directly, soluble CD40 ligand binding to αIIbβ3 induces β3 cytoplasmic tyrosine phosphorylation and fibrinogen binding; the latter response is markedly impaired in platelets carrying non-phosphorylatable β3 cytoplasmic tyrosines, providing evidence that αIIbβ3 outside-in signaling can reinforce receptor function [70]. In addition, αIIbβ3 ligation on immobilized fibrinogen activates Rap1b through a pathway involving Src-family kinases, Ca^2+^, and PKC, thereby returning to a signaling node that also mediates αIIbβ3 inside-out activation [71]. Subsequent studies have shown that cytoskeletal coupling of activated αIIbβ3 through an αIIb-filamin-actin linkage can further stabilize the active integrin state [72], whereas Gα13-dependent outside-in signaling under shear activates a NOX2-ROS-Syk pathway that amplifies secondary platelet activation and thrombus growth [73]. Thus, platelet αIIbβ3 supports the broader concept that ligand engagement and outside-in signaling can reinforce integrin-dependent adhesive responses, although a complete closed feedback loop from outside-in signaling to renewed affinity activation has not been resolved to the same extent as for LFA1.

### 2.3. Common and Context-Specific Features of Integrin Amplification

**Figure 3:**
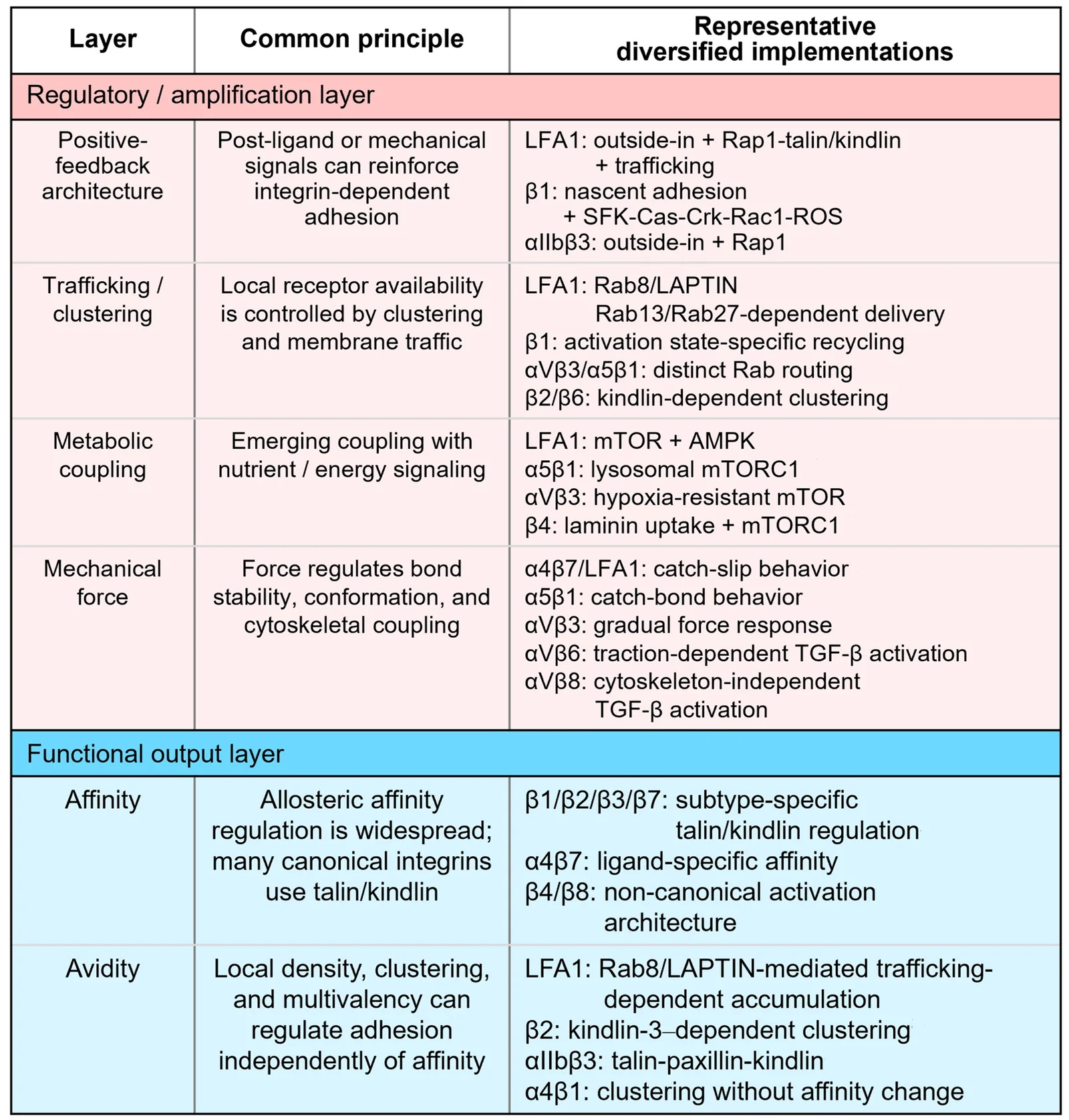
Common principles and representative diversified implementations of integrin regulation. Regulatory/amplification layers (positive-feedback architecture, trafficking/clustering, metabolic coupling, and mechanical force) and functional output layers (affinity and avidity) are organized according to shared system-level principles and representative integrin-specific implementations discussed in the text. The strength of evidence varies among integrin classes; the examples are illustrative rather than exhaustive. Representative studies summarized in the figure are cited in the text [29,32,33,39,43,44,45,61,68,69,71,74,75,76,77,78,79,80,81,82,83,84,85,86,87,88,89,90,91,92,93].

Comparison of adherent-cell adhesions, LFA1, and platelet αIIbβ3 suggests a shared system-level principle of positive-feedback amplification, but not a single conserved molecular circuit. During β1-integrin focal adhesion assembly, an SFK-Cas-Crk-Rac1-ROS positive-feedback loop can precede β1 activation and promote the transition from nascent adhesions containing inactive integrin to mature focal adhesions (Figure 2a) [61]. In LFA1, by contrast, ligand-engaged high-affinity integrin feeds back to the Rap1-talin1-kindlin-3 axis and LFA1 trafficking machinery, thereby coupling affinity and avidity (Figure 1d and Figure 2b) [39,68,69]. Platelet αIIbβ3 provides another implementation in which ligand occupancy and outside-in signaling can influence receptor affinity, Rap1 signaling, cytoskeletal coupling, and ROS-dependent responses [26,70,71,72,73]. Thus, these systems share a common architectural principle in which signals generated during adhesion can reinforce integrin-dependent function through feedback circuits, whereas the initiating integrin state, molecular intermediates, and functional outputs differ among integrins and cellular contexts. This common principle and its representative diversified implementations are summarized in Figure 3. Overall, positive-feedback amplification may therefore represent a general regulatory architecture rather than a universally conserved molecular pathway.

## 3. New Regulators of Outside-In Signaling

The previous section discussed how outside-in signaling can upregulate integrin adhesiveness by reinforcing affinity and avidity. A related question is which molecular modules translate ligand engagement into integrin clustering, contact-site accumulation, and broader cell-state changes. In this section, we focus on the recently identified regulators of integrin outside-in signaling, with an emphasis on LFA1 and on membrane trafficking, ATG8/LC3-associated machinery, metabolic signaling, and mechanical regulation.

### 3.1. Regulators of Clustering and Trafficking

Our recent study identified membrane trafficking and ATG8/LC3-associated machinery as regulators of LFA1 intracellular clustering and contact-site accumulation. LFA1 intracellular clusters colocalize with multiple membrane trafficking factors, including Rab5, Rab7, Rab8, and Rab11 [68,69]. The importance of Rab-mediated integrin trafficking is also supported by several independent studies. Rab13 acts downstream of Mst1 to deliver LFA1 to the cell surface for lymphocyte adhesion and migration [74]. Rab27 regulates the antigen-stimulated translocation of an intracellular LFA1 pool in naïve CD8^+^ T cells [75]. RhoB controls Rab11-mediated recycling and surface reappearance of LFA1 during T-cell migration [94]. Rab35-Arf6 interplay regulates β1-integrin recycling and cell migration in epithelial and cancer-related contexts [95]. Moreover, analyses using BIRT377, which stabilizes low-affinity LFA1, revealed that low-affinity LFA1-dependent outside-in signaling modulates the Rabin8-Rab8 axis to control LFA1 accumulation at the contact plane [68]. In this setting, Rab8-dependent LFA1 trafficking primarily increases the interaction frequency of LFA1 with ICAM1 rather than the lifetime of individual binding events, consistent with avidity modulation rather than affinity maturation (Figure 1d) [68]. Furthermore, EO-dependent LFA1 clusters colocalize with ATG8 family proteins (ATG8s), including the LC3/GABARAP family members [69]. ATG8s are classically known as ubiquitin-like proteins that become lipidated on autophagosomal membranes and contribute to autophagosome biogenesis, maturation, cargo handling, and autophagosome-lysosome fusion [96,97]. Deletion of ATG8s, including LC3B and GABARAP, impairs LFA1 accumulation at the contact plane and LFA1-dependent cell adhesion [69]. Unexpectedly, this function cannot be attributed to canonical degradative macroautophagy, because LFA1-dependent ATG8 clustering and adhesion do not induce autophagic flux [69]. Moreover, analyses of non-canonical autophagy-related markers and the domain requirement of ATG16L1, a scaffold component of the ATG12-ATG5-ATG16L1 machinery that specifies LC3 lipidation in canonical and non-canonical contexts, suggested that this ATG8-related machinery does not simply conform to established non-canonical ATG8 lipidation pathways, such as LC3-associated endocytosis (LANDO) [69,98,99,100]. LANDO and LC3-associated phagocytosis (LAP) are often discussed within a broader family of non-canonical ATG8 lipidation pathways, in which LC3/GABARAP proteins are conjugated to single-membrane endosomes or phagosomes rather than canonical double-membrane autophagosomes [99,100,101,102]. In LANDO, LC3 lipidation occurs on Rab5-positive endosomes and contributes to receptor/cargo trafficking through factors such as Rubicon and ATG16L1, whereas LAP uses related single-membrane LC3 lipidation on phagosomes after cargo engulfment [99,100]. This conceptual background is useful because this LFA1-dependent LC3 lipidation axis also uses ATG8/LC3-associated machinery but differs from LANDO and LAP in being triggered by integrin outside-in signaling and directed toward LFA1 transport and avidity regulation rather than endocytic receptor recycling or phagosome maturation. Based on these distinguishing features, this pathway was defined as a new functional category of ATG8 biology, **L**C3 **A**ssociated **P**rotein **T**ransport induced by **I**ntegri**N** (LAPTIN). The discovery of LAPTIN helps bridge the missing link between outside-in signaling, membrane trafficking, and ligand-induced avidity regulation (Figure 1d and Figure 2b) [69].

Although a LAPTIN-like pathway has not yet been demonstrated for other integrins, several studies indicate that integrin activation state and membrane trafficking are closely coupled. Active and inactive β1 integrins undergo distinct endocytic itineraries: ligand-engaged active β1 integrins are preferentially co-internalized with fibronectin and show slower recycling, whereas inactive β1 integrins undergo rapid Rab4-dependent recycling [76]. More recently, EPLINα was shown to promote the recycling of active β1 integrin from Rab21-positive early endosomes [77]. In addition, membrane tension and caveolae regulate Rab4-dependent recycling and surface availability of active β1 integrin during mechanoadaptation [103]. Growth-factor-regulated routing can also differ between heterodimers: PDGF selectively promotes rapid Rab4-dependent recycling of αVβ3, whereas α5β1 continues through a slower Rab11-dependent route [78]. At the clustering level, subtype-specific adaptor requirements are also evident: chemokine-induced β2-integrin clustering requires kindlin-3 but not talin1 [79], whereas kindlin-2 stabilizes β6-integrin clustering and the active conformation under traction-force-limited conditions [80]. These findings suggest that coupling between integrin activation state and membrane trafficking is not restricted to LFA1, although the molecular machinery and trafficking routes differ among integrin systems, and a LAPTIN-equivalent pathway has not been established in integrins other than LFA1.

### 3.2. Regulation of Metabolism

Alongside the LAPTIN machinery, metabolic signaling has emerged as another layer of LFA1 outside-in regulation. LFA1 EO formation was shown to activate both the mechanistic target of rapamycin (mTOR) and AMP-activated protein kinase (AMPK), two central nutrient- and energy-sensitive kinases [69]. This dual activation is notable because canonical starvation-induced autophagy is typically associated with the AMPK-dependent activation of ULK1 and mTOR-dependent inhibition of ULK1, yielding an AMPK-high/mTOR-low pattern [104]. In contrast, LAPTIN is accompanied by concurrent activation of mTOR and AMPK and does not induce autophagic flux, supporting the view that LAPTIN is not simply a canonical degradative autophagy response redirected to LFA1.

Although direct evidence for mTOR/AMPK signaling downstream of LFA1 outside-in signaling remains limited, related observations in other integrin systems support a broader connection between adhesion and metabolic kinases. In breast cancer cells, vitronectin engagement of αVβ3 integrin maintains mTOR activity and protein synthesis under hypoxic conditions [81]. In endothelial cells, integrin ligation activates ERK through an mTORC2-dependent pathway [105]. Conversely, AMPK has been linked to adhesion control by inhibiting tensin-dependent β1 integrin activity in fibroblasts and by acting as a mechano-metabolic sensor that couples cell adhesion, mitochondrial dynamics, and actomyosin-driven migration in three-dimensional matrices [106,107]. More broadly, these observations fit an emerging view that integrin-mediated adhesion and mechanotransduction are functionally coupled to mTOR signaling in proliferative and invasive cancer cell states [81,108]. In tumor mechanobiology, mTOR signaling components have been discussed as central nodes that integrate extracellular mechanical cues with FAK/Src, PI3K-Akt signaling, cytoskeletal tension, and YAP/TAZ-dependent transcription [108]. These studies indicate that integrin signaling and metabolic sensing are bidirectionally connected, although the molecular logic differs according to the integrin subtype, cell type, and adhesion context. From this perspective, LFA1 outside-in signaling may be regarded not only as an adhesion-strengthening mechanism but also as a local organizer of signaling and metabolic resources at the immune cell contact interface.

More directly linking integrin trafficking to metabolic signaling, ligand-engaged α5β1 integrin undergoes tensin- and Arf4-dependent internalization and trafficking to late endosomes/lysosomes, and this trafficking is required for local recruitment and activation of mTORC1 [82]. In a related nutrient-adaptive pathway, starvation induces β4 integrin and laminin internalization and lysosomal delivery, increasing intracellular amino acid availability and reactivating mTORC1 [83]. Conversely, metabolic sensing can also regulate integrin trafficking, as AMPK activation promotes Arf6/Dab2-dependent clathrin-mediated internalization of β1 integrin [109].

Across these examples, integrin trafficking and metabolic signaling appear to be bidirectionally coupled across several integrin systems, whereas the concurrent activation of mTOR and AMPK associated with LFA1-LAPTIN remains, at present, a distinctive feature of the LFA1 system.

### 3.3. Force-Dependent Amplification

Mechanical force represents another layer of integrin regulation and can amplify integrin-dependent adhesion after ligand engagement. At the single-bond level, the α4β7-MAdCAM1 interaction exhibits catch-slip behavior: moderate tensile force prolongs bond lifetime and stabilizes rolling adhesion, whereas higher force reverses this effect [84]. Similar force-dependent stabilization of ligand-bound integrin states has been demonstrated for α5β1-fibronectin and LFA1-ICAM1 interactions [85,86]. In T cells, F-actin flow is required both to maintain the high-affinity open conformation of LFA1 and to promote its accumulation at the immunological synapse, while direct tension-sensor measurements showed actin-dependent force across the β2 subunit that was associated with active LFA1 and required intact talin- and kindlin-binding motifs [110,111]. More recently, single-molecule analyses demonstrated integrin-subtype–specific force-dependent conformational changes in α5β1 and αVβ3, indicating that force can directly reshape the integrin conformational landscape [87]. On the cytoplasmic side, kindlin-2 converts the mechanically weak talin–β1-integrin slip bond into a force-insensitive ideal bond, supporting sustained force transmission rather than simply increasing talin binding as force rises [58]. The diversity of force coupling is further illustrated by TGF-β-activating αV integrins: αVβ6 uses actomyosin-generated traction to activate latent TGF-β [88], whereas αVβ8 can activate latent TGF-β without cytoskeletal force [89]. Thus, mechanical force may act as a magnitude- and context-dependent amplifier of integrin adhesiveness through stabilization of ligand-bound states, conformational regulation, and reinforcement of cytoskeletal coupling.

### 3.4. Common and Diversified Regulatory Layers of Integrin Adhesiveness

The mechanisms discussed above reveal general regulatory principles as well as substantial integrin-specific diversity (Figure 3). Trafficking and clustering provide a general means of controlling local receptor availability, but the underlying machinery differs among integrins, ranging from Rab8/LAPTIN-dependent LFA1 accumulation to activation-state-specific β1 recycling and subtype-selective Rab or kindlin usage [68,69,76,77,78,79,80]. Metabolic coupling represents an emerging layer: several integrin systems communicate with mTOR, AMPK, or nutrient-acquisition pathways, although the direction and molecular basis of this coupling are highly context-dependent [69,81,82,83,105,106,107,108,109]. Mechanical force likewise regulates integrin-ligand bond stability, receptor conformation, and cytoskeletal coupling, but the magnitude and nature of these responses vary markedly among integrin subtypes [84,85,86,87,88,89,110,111].

These regulatory layers ultimately converge on integrin adhesiveness through affinity, avidity or both, but they need not affect the two parameters equally. LFA1 provides a particularly well-defined example in which ligand-induced outside-in signaling reinforces affinity through the Rap1-talin1-kindlin-3 axis and enhances avidity through trafficking-dependent receptor accumulation [39,68,69]. Affinity regulation itself is diversified: α4β7 can display ligand-selective affinity through conformer-specific activation [90], whereas β4 and β8 lack the canonical talin-binding motifs [91], and αVβ8 displays atypical conformational regulation [92]. Conversely, avidity can be modulated with little or no measurable change in affinity, as shown for rapid α4β1 clustering [93] and β2-integrin clustering [79]. Thus, affinity and avidity can be viewed as common functional outputs produced by diversified regulatory modules rather than by a single universal activation pathway (Figure 3). Not all regulatory layers have been demonstrated to the same extent for every integrin class; the examples in Figure 3 are representative rather than exhaustive.

## 4. Perspectives on Integrin-Targeting Therapeutics

Integrins are clinically validated therapeutic targets for inflammatory and autoimmune diseases, primarily because they control leukocyte adhesion and tissue trafficking. The α4β7-MAdCAM1 axis mediates lymphocyte homing to mucosal tissues [12], whereas α4β1-VCAM1 interactions drive leukocyte entry into the inflamed central nervous system in experimental autoimmune encephalomyelitis and provide a preclinical rationale for α4-integrin blockade in multiple sclerosis [112]. The α4β1-VCAM1 interaction has also been implicated in the rheumatoid synovium [113]. Clinically, LFA1 blockade with efalizumab has shown efficacy in plaque psoriasis [114], selective α4β7 blockade with vedolizumab has demonstrated clinical benefit in ulcerative colitis and Crohn’s disease [115,116], and α4-integrin blockade with natalizumab reduces disease activity in relapsing multiple sclerosis [117]. These examples illustrate that integrin-targeted therapies can suppress pathological leukocyte trafficking and activation while also highlighting the importance of preserving protective immune surveillance.

Natalizumab exemplifies the trade-off between efficacy and safety. As it targets the α4 subunit, it inhibits both the α4β1- and α4β7-dependent trafficking pathways [117]. Although this broad activity contributes to clinical efficacy, it is associated with a risk of JC virus-related progressive multifocal leukoencephalopathy [118,119]. A related limitation is illustrated by efalizumab, an anti-CD11a antibody that blocks LFA1-dependent interactions and was withdrawn after reports of progressive multifocal leukoencephalopathy in patients with psoriasis [120]. These examples do not negate the value of extracellular integrin blockade, but they highlight general limitations of antibodies and ligand-competitive agents. Because many such agents act at or near the ligand-recognition interface, they can suppress both pathogenic and physiological adhesion. Moreover, depending on epitope specificity and valency, antibody binding can stabilize particular integrin conformations or promote receptor clustering, thereby altering outside-in signaling rather than simply blocking it [121]; natalizumab has been reported to elicit direct signaling responses in T cells [122]. Long systemic half-lives, potential immunogenicity, and the difficulty of fine-tuning partial inhibition may further limit their suitability when transient or context-selective modulation is desired.

The integrin outside-in signaling mechanisms discussed in this review raise the possibility of a complementary therapeutic concept, although translating these mechanisms into druggable targets will require extensive validation. Rather than completely preventing ligand binding, it may be possible to tune the amplification phase of integrin activation by targeting intracellular components of outside-in signaling that control adaptor recruitment, clustering, trafficking, and metabolic support. Such an approach could, in principle, dampen excessive integrin function while preserving the basal adhesion required for immune surveillance and maintaining tissue homeostasis. Recognizing both shared and subtype-specific regulatory modules may help identify integrin- or disease-context-selective targets, potentially limiting broad disruption of physiological integrin functions. At present, this concept remains speculative, and candidate axes such as Rab8-dependent transport and LAPTIN-related ATG8 machinery require careful validation for integrin selectivity, druggability, and safety in future studies.

## 5. Outstanding Questions

Several outstanding questions and limitations remain. The evidence should not be overgeneralized. The ligand-first structural kinetics resolved for α5β1 [25] have not yet been reproduced with equivalent temporal and conformational resolution for LFA1 in physiologically activated lymphocytes, and the contribution of outside-in positive feedback may depend on integrin subtype, ligand density, mechanical environment, and cellular context. Similarly, LAPTIN and the associated mTOR/AMPK response are currently best established in the LFA1 system [69]. Determining whether analogous amplification modules operate in other integrins will be important for distinguishing a general principle of integrin biology from specialized mechanisms adapted to leukocyte adhesion. Notably, the available evidence does not support a single conserved amplification mechanism across all integrins. LFA1 provides the clearest example in which ligand engagement feeds back to Rap1-talin1-kindlin-3 and trafficking machinery (Figure 2b and Figure 3), whereas β1 integrin focal adhesions and platelet αIIbβ3 employ partially overlapping but distinct signaling, cytoskeletal, and trafficking modules. Cross-study comparisons are further complicated by differences in cell type, ligand, mechanical environment, and experimental approaches. A separate limitation is that integrin clustering or accumulation is often used as a surrogate for avidity, although these parameters are not necessarily equivalent to direct measurements of adhesive strength.

These uncertainties also define a useful experimental agenda. Future approaches that combine single-molecule measurements of ligand binding and adaptor occupancy with conformational reporters, force-sensitive probes, and real-time trafficking analysis should enable the order of these events to be resolved in intact cells. Comparative analyses of different integrins under matched ligand-density, force, and cellular conditions will also be required to distinguish integrin-specific mechanisms from general principles. Parallel genetic and pharmacological experiments will be needed to distinguish molecules that initiate outside-in amplification from those that merely accompany the stabilized adhesive state. Such mechanistic resolution is also essential for therapeutic translation. If pathological adhesion can be attenuated by selectively weakening positive-feedback amplification rather than abolishing ligand recognition, integrin-targeting strategies may eventually achieve greater context selectivity. The central challenge is therefore to identify which components of the feedback network are sufficiently integrin-proximal and nonredundant to be manipulated without broadly disrupting immune surveillance or other essential cellular functions.

## 6. Conclusions

The emerging picture is that integrin activation cannot always be represented as a linear sequence in which inside-out signaling first generates an active receptor and outside-in signaling begins only after adhesion has been completed. In LFA1, ligand engagement can trigger outside-in signaling from both high- and low-affinity states, with distinct consequences for affinity and avidity regulation, reinforcing affinity through the Rap1–talin1–kindlin-3 axis and avidity through trafficking-dependent integrin accumulation. Evidence from β1-integrin adhesions and platelet αIIbβ3 further suggests that positive-feedback amplification may represent a shared regulatory architecture, although its molecular implementation is strongly integrin- and context-dependent.

Overall, outside-in signaling can function as a detonator-like stimulus that amplifies, rather than merely follows, integrin adhesion, and distinguishing shared regulatory principles from integrin-specific mechanisms may help guide future mechanistic and therapeutic studies.

## Data Availability

No new data were created or analyzed in this study. Data sharing is not applicable to this article.
